# The Role of Melatonin in Salt Stress Responses

**DOI:** 10.3390/ijms20071735

**Published:** 2019-04-08

**Authors:** Junpeng Li, Jing Liu, Tingting Zhu, Chen Zhao, Lingyu Li, Min Chen

**Affiliations:** Shandong Provincial Key Laboratory of Plant Stress Research, College of Life Science, Shandong Normal University, Jinan 250014, China; 18363850217@163.com (J.L.); m15168849563@163.com (J.L.); m15953147572@163.com (T.Z.); 17354609629@163.com (C.Z.); 18753135561@163.com (L.L.)

**Keywords:** plants, salt stress, salt tolerance, melatonin

## Abstract

Melatonin, an indoleamine widely found in animals and plants, is considered as a candidate phytohormone that affects responses to a variety of biotic and abiotic stresses. In plants, melatonin has a similar action to that of the auxin indole-3-acetic acid (IAA), and IAA and melatonin have the same biosynthetic precursor, tryptophan. Salt stress results in the rapid accumulation of melatonin in plants. Melatonin enhances plant resistance to salt stress in two ways: one is via direct pathways, such as the direct clearance of reactive oxygen species; the other is via an indirect pathway by enhancing antioxidant enzyme activity, photosynthetic efficiency, and metabolite content, and by regulating transcription factors associated with stress. In addition, melatonin can affect the performance of plants by affecting the expression of genes. Interestingly, other precursors and metabolite molecules associated with melatonin can also increase the tolerance of plants to salt stress. This paper explores the mechanisms by which melatonin alleviates salt stress by its actions on antioxidants, photosynthesis, ion regulation, and stress signaling.

## 1. Introduction

Environmental stresses can inhibit seed germination, delay growth, promote senescence, and even lead to plant death. Salt stress is a widespread environmental stress factor that seriously restricts agricultural production [1,2,3,4]. Plants respond to salt stress through a variety of biochemical and molecular mechanisms, which act at the cellular and whole-plant levels [5]. The strategies at the cellular level include the selective absorption and exclusion of ions, compartmentalization of ions into the central vacuole, the synthesis and accumulation of organic solutes in the cytoplasm, and changes in membrane composition [2,5,6,7]. At the whole-plant level, the strategies include control of the ion absorption by roots, control of the ion transport from roots to shoots, distribution of ions in shoots to different organs (e.g., old leaves and leaf sheathes), changing the photosynthetic pathway, modifying the activity of antioxidant enzymes, and altering the levels of plant hormones [4,8,9,10]. Plant hormones such as ethylene [11], jasmonic acid [12], gibberellic acid [13], and abscisic acid [14], as well as a number of other molecules, such as nitric oxide [15,16], hydrogen sulfide [17], and calcium [18,19,20], are involved in plant response to salt stress. In addition, numerous studies have shown that melatonin plays an important role in the response of plants to salt stress.

Melatonin, *N*-acetyl-5-methoxy-tryptamine, was first identified in 1958 and was named for its function in reversing the darkening effect of melanocyte-stimulating hormone [21]. Early studies of melatonin concentrated on its function in animals. Experiments in animals showed that melatonin plays important roles in regulating the activities of antioxidant enzymes [22], circadian rhythms [23], physical conditions, emotional status, and the effects of some diseases, such as coronary heart disease and Alzheimer’s disease [24,25,26]. Melatonin was first discovered in plants in 1995 [27]. Subsequently, melatonin has been found in different plant species and in various organs, such as roots, stems, leaves, fruits, and seeds. In plants, melatonin functions as a metabolite with multiple functions, including the response to plant stresses such as salinity [28], cold [29], drought [30], reactive oxygen species (ROS) [31], and nutritional deficiency [32]; it also regulates growth and development, such as root organogenesis, flowering, and senescence [33,34,35,36,37]. In recent years, many studies have focused on the function and regulation of melatonin in plants adapted to salt stress. For soybean (*Glycine max*) plants, seeds soaked with melatonin grow into robust seedlings with larger leaves, higher plant height and biomass, and more pods and seeds than that without melatonin treatment. At the same time, melatonin treatment increased the salt and drought tolerance of the soybean plants, so application of melatonin can improve field crops in this species [38]. Similar results were obtained in bermudagrass (*Cynodon dactylon*), and exogenous melatonin treatment increased its salt, drought, and cold tolerance compared with untreated plants [39]. Many results showed that melatonin-treated plants have stronger salt tolerance and further experimental results indicate that melatonin-treated plants have lower ROS, electrolyte leakage, and cell damage while having greater plant height, biomass, and organic matter content than untreated plants [40]. Similar results were also found in salt-stressed plants, such as *Citrus aurantium* seedlings [41], sunflower [42], and cucumber [43]. This review summarizes the mechanisms by which melatonin alleviates salt damage and discusses the melatonin-mediated regulation of growth and development and salt stress signaling in plants.

## 2. Melatonin Biosynthesis in Plants

**Figure 1:**
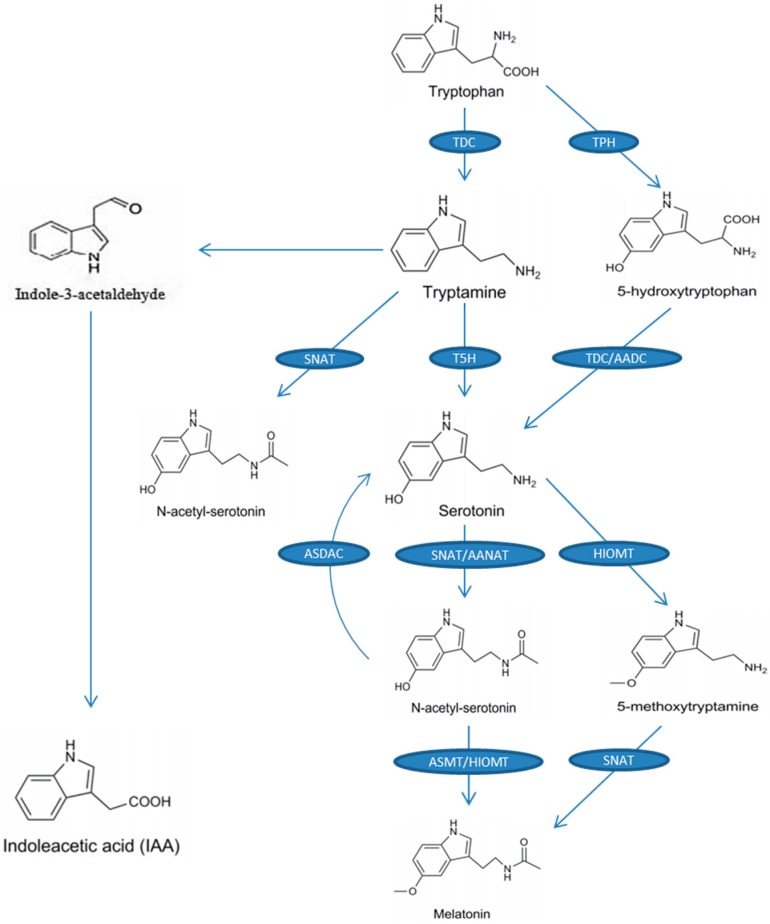
Biosynthetic pathway of melatonin from tryptophan in plants. The enzymes of the respective steps are as follows: TDC: tryptophan decarboxylase; T5H: tryptamine 5-hydroxylase; SNAT: serotonin-*N*-acetyltransferase; AANAT: arylalkylamine *N*-acetyltransferase; ASMT: *N*-acetylserotonin methyltransferase; HIOMT: hydroxyindole-*O*-methyltransferase; AADC: aromatic-L-amino-acid decarboxylase; TPH: tryptophan hydroxylase; ASDAC: *N*-acetylserotonin deacetylase; IAA: indole-3-acetic acid.

The precursor of melatonin is tryptophan. The entire biosynthetic process (from tryptophan to melatonin) requires four enzymatic reactions (Figure 1). The first enzyme is tryptophan decarboxylase (TDC), which catalyzes the conversion of tryptophan to tryptamine, and then tryptamine 5-hydroxylase (T5H) catalyzes the conversion of tryptamine to serotonin [44], which is the main biosynthetic pathway of serotonin in plants. Another serotonin biosynthetic pathway exists in some plants, such as St. John’s wort (*Hypericum perforatum*), and is similar to the melatonin biosynthesis pathway in animals, in which tryptophan 5-hydroxylase (TPH) converts tryptophan to 5-hydroxytryptophan and then 5-hydroxytryptophan is catalyzed into serotonin by aromatic-l-amino-acid decarboxylase (TDC/AADC) [45]. Serotonin is then converted into *N*-acetyl-serotonin by the catalytic reaction of *N*-acetyltransferase (SNAT) or arylalkylamine *N*-acetyltransferase (AANAT) and then *N*-acetyl-serotonin is converted to melatonin by *N*-acetyl-serotonin methyltransferase (ASMT) or hydroxyindole-*O*-methyltransferase (HIOMT). Additionally, SNAT can catalyze tryptamine to *N*-acetyl-tryptamine, but T5H cannot further convert *N*-acetyl-tryptamine into *N*-acetyl-serotonin [46]. To our knowledge, no pathway has been found for the conversion of *N*-acetyl-tryptophan to *N*-acetyl-serotonin. Serotonin can also be converted to 5-methoxytryptamine by HIOMT and, finally, 5-methoxytryptamine is catalyzed by SNAT to melatonin [47]. Figure 1 shows the biosynthesis of melatonin and the chemical structure of melatonin and each intermediate.

A recent study revealed a reverse melatonin pathway, in which *N*-acetyl-serotonin deacetylase catalyzes *N*-acetyl-serotonin into serotonin [48]. In addition to being the precursor for melatonin, tryptophan is also the precursor for indole-3-acetic acid (IAA; a kind of phytohormone). One of the pathways of IAA synthesis is the tryptamine pathway, and in this pathway tryptophan is catalyzed into tryptamine, and then tryptamine is converted to IAA by indole-3-acetaldehyde as an intermediate (Figure 1) [49]. This indicates that melatonin may have similar effects in plants to those of IAA. A recent study suggested a possible, previously unknown, action for melatonin in plants, whereby indoleamine promotes vegetative growth in etiolated lupin (*Lupinus albus* L.) hypocotyls in a similar manner to IAA [35]. Of particular note was the inhibitory growth effect of melatonin on the monocot roots assayed, which was similar to that of IAA [50].

## 3. Endogenous Melatonin in Plants

Melatonin is thought to be synthesized in mitochondria and chloroplasts of plants [51], and has been found to be widespread in a variety of plants, including herbs, agricultural crops, fruit trees, etc. [52,53,54,55,56,57]. Melatonin levels in plants may be subjected to circadian and seasonal rhythms, so some of the published levels given in various plants may be only approximate [58]. Although the melatonin content varies greatly among different plant species, the distribution of endogenous melatonin in different organs and developmental periods of different plants is similar [53]. In addition to circadian and seasonal rhythms, stress and stages of development also affect melatonin levels in plants. In morning glory (*Pharbitis nil* Choisy) and tomato (*Lycopersicon esculentum* Mill.), the melatonin content usually increases during maturation [54].

Recent studies showed that environmental conditions such as light affect the levels of endogenous melatonin in plants. Melatonin levels in tomato plants grown in an open field were higher than those in plants cultured in chambers [55]. The melatonin levels in senescing rice (*Oryza sativa* cv. Asahi) leaves under constant illumination were higher than those in constant darkness, which suggested that light signals induce melatonin biosynthesis during leaf senescence [56]. However, another study showed that the melatonin content in grapevine (*Vitis vinifera*) dropped dramatically during the day and was the highest in the dark, which indicated that light inhibits the synthesis of melatonin in this species [57].

## 4. Stress-Induced Melatonin Accumulation

In plants, melatonin serves as a unique antioxidant, and can interact with ROS and reduce their levels under stress [57]. Thus, under stress conditions, the increase of melatonin content is related to the increase of ROS level [59]. The content of melatonin in the roots and leaves of grapevine seedlings increased significantly under salt stress, and the increase was enhanced with the intensity of the stress [60]. The melatonin levels in barley (*Hordeum vulgare* L.) were significantly increased after osmotic stress, and the same result was observed in lupin [61]. The melatonin content in rice seedlings increased at high temperature [62]. These data indicate that the biosynthesis of endogenous melatonin is induced by stress conditions, which indicates that the molecule plays a role in the plant’s response to various stresses [63]. The accumulation of melatonin in plants is closely related to the expression of genes and the activity of enzymes associated with melatonin biosynthesis and catabolic pathways. For example, the high expression of genes encoding melatonin synthetases (e.g., TDC, T5H, ASMT) in rice grown with excess cadmium (Cd) was found to be closely related to melatonin levels [64]. In addition, increased production of melatonin in rice is associated with the increased enzymatic activities of SNAT and ASMT under high temperature [62].

Generally, melatonin concentration is closely connected to the availability of its precursors [63,65], and serotonin plays a role in the stress response of rice under cold conditions [66]. Higher levels of 2-hydroxymelatonin in rice under cold and arid conditions suggest a potential role in resistance to these stresses [62,67]. The concentration of melatonin was increased in tomato via direct binding of a transcription factor (HsfA1a) to the caffeic acid *O*-methyltransferase 1 (*COMT1*) gene promoter under Cd stress [68]. Endogenous serotonin and melatonin content increased in roots and cotyledons under NaCl stress, indicating their involvement in salt stress. Further analysis found that NaCl stress modulates the activity of *N*-acetyl-serotonin *O*-methyltransferase (ASMT), the enzyme responsible for melatonin biosynthesis from *N*-acetyl-serotonin [42]. However, the mechanism by which the melatonin biosynthesis pathway is regulated in response to other stresses remains unclear.

## 5. Functions of Exogenous Melatonin under Salt Stress

### 5.1. Effect of Melatonin as an Auxin on Plant Growth and Development

Melatonin is a kind of indoleamine, and it shares the same biosynthetic precursor with IAA, so melatonin and IAA are structurally similar (Figure 1). Therefore, melatonin may regulate plant growth and development in a similar manner to that of IAA. Studies have found that melatonin is an auxinic hormone in monocots such as canary grass (*Phalaris canariensis*), barley, wheat (*Triticum* sp.), and oat (*Avena sativa*) [50], and in dicots such as *Arabidopsis thaliana*, lupin (*Lupinus micranthus* Guss.) [69,70]. When melatonin level is low in plants, exogenous melatonin has a significant effect on plant growth and development [71]. Exogenously applied melatonin is permeable across the plasma membrane and increases the endogenous melatonin concentration, which was shown to promote soybean plant growth and seed yield [38]. Kolář et al. (2003) reported that exogenous melatonin plays a role in the early stages of flower development regulated by photoperiod [34]. Zhang (2014) reported that exogenously applied melatonin promoted the formation of lateral roots of cucumber (*Cucumis sativus*) [72]. When applying exogenous melatonin to plants, a suitable melatonin concentration is necessary, and the optimum concentration of exogenous melatonin is different for different plants. In *Arabidopsis* seedlings, a moderate melatonin concentration (40 µM) promotes plant growth and development, a low concentration (10–20 µM) has no obvious effect, and a high concentration (200–400 µM) inhibits plant growth [29].

Salt stress not only inhibits many plant physiological processes, but also induces large amounts of ROS [73,74,75,76]. So, up-regulating melatonin biosynthesis is necessary for reducing the ROS injury to plants [77,78]. At the same time, the application of exogenous melatonin to plants is considered to be an effective means of ameliorating salt stress. Melatonin can promote root development, which ensures that the plant has a strong root system. The stress resistance of plants is directly related to strong roots. For example, exogenous applications of melatonin promote the regeneration of lateral roots and adventitious roots in etiolated hypocotyls of lupin and black mustard seed (*Brassica juncea*) [79,80]. Under high salt conditions, seed germination and root elongation were inhibited, plant growth was decreased, and net photosynthetic rate and chlorophyll content were decreased, whereas pre-treatment with exogenous melatonin allowed plants to maintain robust roots, reduce growth inhibition, and improve photosynthetic capacity [77]. Salt stress affects the content of some plant hormones, such as gibberellin (GA) and abscisic acid (ABA), inhibiting plant growth [81,82,83]. Exogenous application of melatonin can increase the salt tolerance of cucumber by mediating the expression of the genes associated with the biosynthesis and catabolism of GA and ABA [43]. Further studies have found that the exogenous application of melatonin down-regulates the ABA biosynthetic genes and up-regulates the ABA catabolic genes, which decreases the ABA content and promotes the growth of soybean under salt stress [38]. For GA, exogenous melatonin up-regulates the expression of key GA biosynthesis genes (*GA20ox* and *GA3ox*) and causes an increase in GA content, which increases the seed germination rate under salt stress [43].

Not only melatonin but also melatonin precursors, its related intermediates, and metabolites have been found to be involved in plants’ response to stress tolerance [84,85]. Tryptophan is a substrate for auxins, indoles, alkaloids, glucosinolates, and phytoalexins—important molecules in the plant stress response [45]. Tryptamine is closely connected with light-enhanced resistance to *Magnaporthe grisea* in rice [86]. Serotonin can enhance the resistance to salt stress by regulating the flow of ions into the chloroplast [87]. *N*-acetylserotonin has antioxidant activity in animals, but its involvement in plants’ stress response has not yet been identified [88]. Based on their molecular similarity to melatonin, 5-methoxytryptamine, cyclo-3-hydroxy melatonin, and AFMK are considered to be involved in plant stress tolerance, but have yet to be explored.

### 5.2. The Antioxidative Function of Melatonin

Salt stress leads to an increase in reactive oxygen species, which on the one hand cause cell damage and on the other hand induce protective responses [4,89,90,91,92,93]. The ROS scavenging system in plants consists of non-enzymatic antioxidants and enzymatic antioxidants; the former includes melatonin and classic antioxidants such as vitamin C, vitamin E, and glutathione. Melatonin exhibits a more potent antioxidant capacity compared to other non-enzymatic antioxidants [51]. Endogenously produced and exogenously applied melatonin can effectively reduce oxidative injury produced by ROS. It is estimated that melatonin scavenges ROS via the cascade reaction [51]. Studies showed that melatonin-pretreated plants had a relatively low H_2_O_2_ content. Therefore, the antioxidant function of melatonin has attracted attention, and it is generally believed that its main role is to directly scavenge ROS.

Enzymatic antioxidants also provide a highly efficient and specific ROS scavenging system for plants, including superoxide dismutase (SOD), catalase (CAT), ascorbate oxidase (APX), glutathione peroxidase (GPX), and glutathione reductase (GR). These antioxidant enzymes tend to increase when plants are under salt stress, and their levels are related to the salt tolerance of the plant [94,95]. Studies showed that melatonin-pretreated seedlings had higher antioxidant enzyme activities than untreated seedlings [96]. Likewise, it is generally believed that melatonin can increase the activity of antioxidant enzymes. In addition, melatonin can increase the efficiency of the mitochondrial electron transport chain, thereby easing electron leakage and reducing the generation of free radicals, which in turn protects antioxidant enzymes from oxidative damage [97].

### 5.3. Melatonin Promotes Photosynthesis under Salt Stress

Melatonin has an important function in photosynthesis and photoprotection [37]. Salt stress limits the absorption of light energy and electron transport in photosystem II (PSII) by decreasing the chlorophyll content, the actual photochemical efficiency of PSII, and photochemical quenching (qP) [98], which has an adverse effect on the bioenergetic process of photosynthesis [76,99]. Melatonin treatment reduced the inhibition of photosynthesis and biomass caused by salt stress. Melatonin has protective effects on chlorophyll, which was discovered in the macroalga *Ulva* sp. [100] and in the freshwater *Chara australis,* which showed that melatonin protects chlorophyll and increases the efficiency of the reaction centers of photosystem II [101]. Recently, similar data have been obtained in salt-stressed bermudagrass, citrus, and sunflower, which confirmed the protective role of melatonin on the photosynthetic pigments [40].

Plants close their stomata under salt stress to reduce water loss, so stomatal conductance (GS) is reduced, which in turn reduces photosynthesis [102,103]. However, the use of an optimal dose of melatonin can improve stomatal function and enable plants to reopen their stomata under salt stress [104]. The tendency of the net photosynthetic rate (Pn) to rapidly decrease under salt stress is ameliorated by the application of melatonin. Melatonin treatment improved the maximum photochemical efficiency of PSII (Fv/Fm) and the total chlorophyll content by enhancing the biosynthesis of chlorophyll and slowing the rate of its decomposition under salt stress. Therefore, melatonin plays a key role in protecting PSII and ameliorating the decrease of chlorophyll content under salt stress. In bermudagrass (*Cynodon dactylon*), melatonin upregulated the expression of photosynthesis-related genes under salt stress and had a positive effect on glucose metabolism, fatty acid metabolism, and ascorbic acid synthesis [40]. The gene expression levels of the subunits PsbO and PsbP of the photosystem I (PSI)-related proteins PsaK and PsaG and the PSII photochemical reaction center protein OEC (oxygen-evolving enhancer proteins) were up-regulated under melatonin treatment. Moreover, under salt stress, melatonin increased the transcription level of photosynthesis-related genes and protected the photosynthetic apparatus [105].

### 5.4. Effect of Melatonin on Ion Regulation and Compartmentalization

Ion uptake and compartmentalization is important for salt tolerance in plants because excessive salt ions in the cytoplasm disrupt ion homeostasis and inhibit plant growth and development [106,107,108,109]. Therefore, under high saline conditions, plants move excessive salt ions in the cytoplasm into the vacuole or compartmentalize them into different tissues [81,110,111]. The salt-induced Na^+^/H^+^ antiporter located in the tonoplast is responsible for compartmentalizing ions in the cytoplasm into the vacuoles to decrease the ion levels in the cytoplasm [112,113]. Melatonin plays a key role in maintaining ion homeostasis. The salt tolerance of M.26 (an important dwarf rootstock of apple (*Malus domestica*)) was enhanced by melatonin via up-regulation of *MdNHX1* (*Malus* vacuolar Na^+^/H^+^ antiporter gene) [114]. The inward-rectifying channel AKT1 (*Arabidopsis* K^+^ transporter 1) mediates the relative uptake rates of Na^+^ and K^+^ under high salinity [115,116] and in M.26, *MdAKT1* (*Malus* inward-rectifying channel AKT1) was observed to have the same effect, and was highly expressed in leaves. Thus, melatonin alleviates the damage caused by high-salt conditions by maintaining ion homeostasis via modulating the expression of *MdNHX1* and *MdAKT1* in apple. Shi and Zhu (2002) reported that salt stress and ABA regulate the tissue distribution and expression level of *AtNHX1* [117,118]. Studies have found that treatment with NaCl or ABA can up-regulate the steady-state levels of *AtNHX1* transcripts. Moreover, the up-regulation of *AtNHX1* expression under salt stress is partially dependent on ABA biosynthesis and ABA signaling through ABI1 (ABA-insensitive pathway 1) [117]. However, the application of melatonin under salt stress can affect the decomposition and synthesis of ABA, and it is suspected that melatonin affects ion regulation and partitioning through the ABA biosynthesis and signaling pathway. The exact function of melatonin under salt stress is that melatonin maintains ion homeostasis by up-regulating the transporter genes *NHX1* and *AKT1* [28]; however, elucidation of the mechanism of salt tolerance induced by melatonin under salt stress requires further investigation.

### 5.5. Melatonin Modulates the Activity of Transcription Factors

One of the key ways in which melatonin regulates the salt tolerance of plants is by modulating the activity of transcription factors. The main melatonin-mediated transcription factors in plants are zinc finger protein 6 (ZAT6), heat shock factors (HSFA1s), and C-repeat-binding factor/drought response element binding 1 factors (CBF/DREB1s). At the same time, multiple stress response genes (*cold-inducible 1* (*KIN1*), *cold-related 15A* (*COR15A*), and *responsive to dehydration 22* (*RD22*)) are up-regulated by *CBF/DREB1*, which is closely related to high levels of melatonin and thus increases plant resistance to salt, drought, and freezing stresses [119]. Melatonin-activated transcription factors regulate the transcription of stress-responsive genes to resist abiotic stresses. Furthermore, 2-hydroxymelatonin, a metabolite of melatonin in rice, upregulates the transcription factors Myb4 and AP37 in response to a variety of abiotic stresses [66]. In apple, salt stress leads to chlorophyll degradation and leaf senescence in leaves, but exogenous melatonin suppresses the transcript levels of a key chlorophyll degradation gene, *pheophorbide a oxygenase* (*PAO*), *senescence-associated gene 12* (*SAG12*), and *auxin resistant 3 (AXR3)*/*indole-3-acetic acid inducible 17 (IAA170)*, which relieves the chlorophyll degradation and leaf senescence caused by salt stress. Therefore, melatonin plays important roles in slowing the senescence of plant leaves [120]. Similar results were found in cucumber roots under NaCl stress, in which melatonin up-regulated 77 differentially expressed genes, including some important transcription factors (e.g., MYB, WRKY, NAC, and ERF). Up-regulation of these transcription factors is closely related to the salt tolerance of cucumber [121]. These results indicate that melatonin can increase the salt tolerance of plants by up-regulating the expression of related transcription factors. However, no studies have reported the unique signaling pathway of melatonin. Therefore, the focus of future work will be to elucidate the signaling pathway of melatonin.

## 6. Conclusions and Perspectives

**Figure 2:**
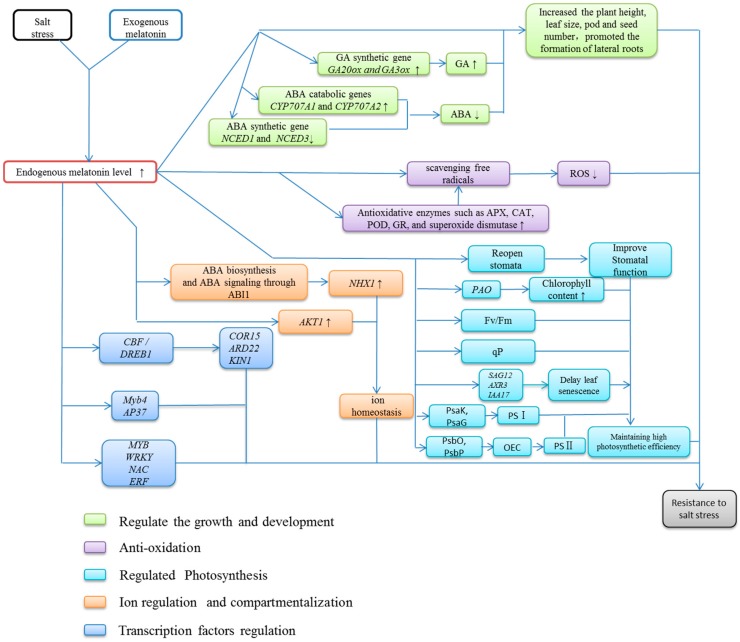
Overview of plant stress responses to exogenous melatonin treatment under salt stress. Melatonin promotes plant growth, regulates photosynthesis, maintains ion homeostasis, and alters the expression of stress-related genes. Different colored boxes represent different effects of melatonin on plant stress response under salt stress. ↑ represents promotion; ↓ represents suppression. ABA: abscisic acid; ABI: ABA-insensitive; AKT1: *Arabidopsis* K^+^ transporter; AP37: APETALA 37, a MADS box transcription factor; APX: ascorbate peroxidase; AXR3: auxin-resistance gene 3; CAT: catalase; CBF: C-repeat-binding factor; COR15A: cold-related 15A; CYP707A: the key enzymes of the ABA 8′-hydroxylation reaction, all are members of the cytochrome P450 (CYP) superfamily; DREB1: drought response element binding 1 factors; ERF: ethylene response factor; Fv/Fm: maximal photochemical efficiency; GA: gibberellins; GA20ox: GA20-oxidase; GA3ox: GA3-β hydroxylase; GR: glutathione reductase; IAA17: Aux/IAA gene family 17; KIN1: cold-inducible 1; MYB: myeloblastosis, a transcription factor family; Myb4: MYB transcription factor family 4; NAC: NAM, ATAF1,2, CUC2; NCED: nine-*cis*-epoxycarotenoid dioxygenase; NHX: Na^+^/H^+^ antiporter; OEC: oxygen-evolving complex; PAO: pheophorbide a oxygenase; POD: peroxidase; PSI: photosystem I; PSII: photosystem II; PsaG: PSI complex small subunit G; PsaK: PSI complex small subunit K; PsbO: PSI complex small subunit O; PsbP: PSI complex small subunit P; qP: photochemical quenching; RD22: responsive to dehydration gene 22; ROS: reactive oxygen species; SAG12: senescence-associated genes 12; WRKY: all transcription factors contain a conserved WRKYGQK domain.

Significant progress has been made in understanding the roles of melatonin in plants. These studies have further expanded our knowledge of the levels and distribution of melatonin, its metabolism, and its function in plants. However, the signaling pathway of melatonin under salt stress remains unclear. The level of melatonin increases substantially in plants under salt stress, which is believed to play important roles in stress resistance. Exogenous application of melatonin ameliorates the deleterious effects of salt stress. However there is currently no systematic description of the role of melatonin under salt stress. As a multifunctional factor, melatonin can regulate plant growth and stress resistance. Suitable concentrations of melatonin can promote growth and maintain high vigor in *Arabidopsis* seeds under salt stress [122]. Melatonin reduces salt damage mainly through (1) reducing excessive ROS [123], (2) plant growth [124], (3) regulating ion homeostasis [28], and (4) modulating the activity of transcription factors [121] (Figure 2). It has been confirmed that melatonin is a key molecule in a very efficient antioxidant cascade and shows similarity to the IAA molecule [125]. Other effects of melatonin are uncertain and require more experimental validation.

### 6.1. Exploiting the Mechanism behind the Melatonin-Mediated Increase of Antioxidant Enzyme Activity

The activities of antioxidant enzymes (SOD, CAT, APX, GPX, and GR) in plants are closely related to plant salt tolerance. Studies have shown that melatonin-pretreated seedlings have higher antioxidant enzyme activities than untreated seedlings [96], which showed that exogenous applications of melatonin can increase antioxidant enzyme activities, but the specific mechanism behind this is not clear. The aim of future research will be to exploit the key mechanism by which melatonin increases antioxidant enzyme activities.

### 6.2. Exogenous Applications of Melatonin Promote Plant Growth and Increase Salt Tolerance

Melatonin treatment reduced the inhibition of photosynthesis and biomass caused by salt stress. The application of exogenous melatonin can improve stomatal function, increase the total chlorophyll content and net photosynthetic rate, improve the maximum photochemical efficiency of PSII, and up-regulate the expression of genes related to the photosynthesis dark reaction [103,104,105], which promotes plant growth and increases salt tolerance.

### 6.3. Application of Exogenous Melatonin Regulates Ion Homeostasis in Plants under Salt Stress

Melatonin modifies the activities of the inward-rectifying channel AKT1 and the Na^+^/H^+^ antiporter (located in the tonoplast) to control the relative uptake rates of Na^+^ and K^+^ and compartmentalize ions into the vacuoles, which decreases the ion levels in the cytoplasm and reconstitutes ion homeostasis [28,114]. However, the specific mechanism behind this is not clear. The aim of future research will be to exploit the mechanism of melatonin-mediated ion homeostasis in plants under salt stress.

### 6.4. Melatonin Is Involved in Regulating Transcription Factors Related to Stress

In plants, melatonin-mediated stress-related transcription factors are zinc finger protein 6 (ZAT6), heat shock factors (HSFA1s), and C-repeat-binding factor/drought response element binding 1 factors (CBF/DREB1s). At the same time, multiple stress response genes (cold-inducible 1 (*KIN1*), cold-related 15A (*COR15A*), and responsive to dehydration 22 (*RD22*)) are up-regulated by *CBF/DREB1* [126]. It is currently unknown how these up-regulated transcription factors affect the salt tolerance of plants. Therefore, the next step is to elucidate the signaling pathway of melatonin.

This review discusses the roles of melatonin in salt resistance in plants and lays the foundation for further study of the melatonin-related salt resistance mechanism. We summarize the biosynthesis of melatonin, its response to stress, its roles in stress resistance, and possible mechanisms. Plants synthesize melatonin and accumulate high levels of melatonin under salt stress. The application of exogenous melatonin also plays an important role in resisting salt stress. However, more research is needed to better understand the metabolism and regulation pathways of melatonin to take advantage of these functions.
